# Selective photoinactivation of *Candida albicans* in the non-vertebrate host infection model *Galleria mellonella*

**DOI:** 10.1186/1471-2180-13-217

**Published:** 2013-10-01

**Authors:** José Chibebe Junior, Caetano P Sabino, Xiaojiang Tan, Juliana C Junqueira, Yan Wang, Beth B Fuchs, Antonio OC Jorge, George P Tegos, Michael R Hamblin, Eleftherios Mylonakis

**Affiliations:** 1 Department of Biosciences and Oral Diagnosis, Univ Estadual Paulista/UNESP, São José dos Campos, SP 12245000, Brazil; 2 Division of Infectious Diseases, Massachusetts General Hospital, Boston, MA 02114, USA; 3 Department of Restorative Dentistry, Faculty of Pindamonhangaba, Pindamonhangaba, SP 12422970, Brazil; 4 Wellman Center for Photomedicine, Massachusetts General Hospital, Boston, MA 02114, USA; 5 Center for Lasers and Applications, Nuclear and Energy Research Institute, São Paulo, SP 05508000, Brazil; 6 Huiqiao Department, Nanfang Hospital, Southern Medical University, Guangzhou 510515, People’s Republic of China; 7 School of Pharmacy, Second Military Medical University, Shanghai 200433, China; 8 Department of Pathology and Center for Molecular Discovery, University of New Mexico, Albuquerque, NM 87131, USA; 9 Department of Dermatology, Harvard Medical School, Boston, MA 02114, USA; 10 Harvard-MIT Division of Health Sciences and Technology, Cambridge, MA 02139, USA; 11 Warren Alpert Medical School, Brown University/Rhode Island and Miriam Hospitals, Providence, RI 02903, USA

**Keywords:** *Candida albicans*, Photodynamic therapy, *Galleria mellonella*

## Abstract

**Background:**

*Candida* spp. are recognized as a primary agent of severe fungal infection in immunocompromised patients, and are the fourth most common cause of bloodstream infections. Our study explores treatment with photodynamic therapy (PDT) as an innovative antimicrobial technology that employs a nontoxic dye, termed a photosensitizer (PS), followed by irradiation with harmless visible light. After photoactivation, the PS produces either singlet oxygen or other reactive oxygen species (ROS) that primarily react with the pathogen cell wall, promoting permeabilization of the membrane and cell death. The emergence of antifungal-resistant *Candida* strains has motivated the study of antimicrobial PDT (aPDT) as an alternative treatment of these infections. We employed the invertebrate wax moth *Galleria mellonella* as an *in vivo* model to study the effects of aPDT against *C. albicans* infection. The effects of aPDT combined with conventional antifungal drugs were also evaluated in *G. mellonella*.

**Results:**

We verified that methylene blue-mediated aPDT prolonged the survival of *C. albicans* infected *G. mellonella* larvae. The fungal burden of *G. mellonella* hemolymph was reduced after aPDT in infected larvae. A fluconazole-resistant *C. albicans* strain was used to test the combination of aPDT and fluconazole. Administration of fluconazole either before or after exposing the larvae to aPDT significantly prolonged the survival of the larvae compared to either treatment alone.

**Conclusions:**

*G*. *mellonella* is a useful *in vivo* model to evaluate aPDT as a treatment regimen for *Candida* infections. The data suggests that combined aPDT and antifungal therapy could be an alternative approach to antifungal-resistant *Candida* strains.

## Background

*Candida albicans* and other *Candida* species commonly colonize the epithelial surfaces of the human body [1]. One-half of humans have oral cavities colonized by *Candida* species in a commensal relationship with the host [2]. Although few healthy carriers develop clinical candidiasis, when the host becomes immunocompromised due to cancer, HIV/AIDS, diabetes, major surgery, transplantation of solid organs or hematopoietic stem cells, these opportunistic pathogens can cause superficial infections that may be cutaneous, subcutaneous or mucosal. In progressive cases, the fungus can penetrate the epithelial surface and be disseminated by the bloodstream with serious consequences [1,3-7].

*C. albicans* is the most common species isolated from superficial and systemic candidiasis and it is considered the most pathogenic species of the *Candida* genus [5,8-11]. *In vitro* investigations indicate that *C. albicans* expresses higher levels of putative virulence factors compared to other *Candida* species. It has been proposed that several virulence factors are involved in the pathogenicity of *C. albicans*, such as adhesion to host surfaces, hyphal formation and secretion of proteinases [11]. In addition, *C. albicans* cells employ mechanisms that protect of the fungal cells from the host immune system, including an efficient oxidative stress response [12,13]. When immunocompetent individuals are infected by fungi, macrophages and neutrophils generate reactive oxygen species (ROS), such as superoxide radicals and hydrogen peroxide that damage cellular components of *C. albicans*, inclusive of proteins, lipids and DNA. The production of ROS is an important mechanism of host defense against fungal pathogens [13], damaging cells enough to cause cell death of phagocytosed fungal cells [12,14].

Treatment of fungal infections, especially invasive ones, is considered difficult due to the limited availability of antifungal drugs and by the emergence of drug-resistant strains. The development of new antifungal agents and new therapeutic approaches for fungal infections are therefore urgently needed [4,8,15]. Photodynamic therapy (PDT) is an innovative antimicrobial approach that combines a non-toxic dye or photosensitizer (PS) with harmless visible light of the correct wavelength. The activation of the PS by light results in the production of ROS, such as singlet oxygen and hydroxyl radicals, that are toxic to cells [6,16]. PDT is a highly selective modality because the PS uptake occurs mainly in hyperproliferative cells and cell death is spatially limited to regions where light of the appropriate wavelength is applied. As microbial cells possess very fast growth rates, much like that of malignant cells, PDT has been widely used for microbial cell destruction [17]. Several *in vitro* studies have shown that PDT can be highly effective in the inactivation of *C. albicans* and other *Candida* species. Therefore, antifungal PDT is a subject of increasing interest especially against *Candida* strains resistant to conventional antifungal agents [16].

*Galleria mellonella* (the greater wax moth) has been successfully used to study pathogenesis and infection by different fungal species, such as *Candida albicans*, *Cryptococcus neoformans*, *Fusarium oxysporum*, *Aspergillus flavus* and *Aspergillus fumigatus*[18]. Recently, our laboratory was the first to describe *G. mellonella* as an alternative invertebrate model host to study antimicrobial PDT alone or followed by conventional therapeutic antimicrobial treatments [19]. We demonstrated that after infection by *Enterococcus faecium*, the use of antimicrobial PDT prolonged larval survival. We have also found that aPDT followed by administration of a conventional antibiotic (vancomycin) was significantly effective in prolonging larval survival even when infected with a vancomycin-resistant *E. faecium* strain.

In this study, we go on to report the use of the invertebrate model *G. mellonella* as a whole animal host for the *in vivo* study of antifungal PDT, as well as the study of combined therapy using PDT and a conventional antifungal drug.

## Methods

### Microbial strains and culture conditions

The *C. albicans* strains used in this study were Can14 and Can37*. C. albicans* Can14 is a wild-type strain SC5314 [20] and *C. albicans* Can37 is a fluconazole resistant clinical isolate from a patient with oropharyngeal candidiasis [3]. *C. albicans* Can37 was identified by growth on Hicrome *Candida* (Himedia, Munbai, India), germ tube test, clamydospore formation on corn meal agar, and API20C for sugar assimilation (BioMerieux, Marcy Etoile, France). Susceptibility pattern to fluconazole was determined by the broth microdilution assay according to the Clinical and Laboratory Standards Institute (CLSI).

Strains were stored as frozen stocks with 30% glycerol at -80°C and subcultured on YPD agar plates (1% yeast extract, 2% peptone, and 2% dextrose) at 30°C. Strains were routinely grown in YPD liquid medium at 30°C in a shaking incubator.

### Fungal inocula preparation

*C. albicans* cells were grown in YPD at 30°C overnight. Cells were collected with centrifugation and washed three times with PBS. Yeast cells were counted using a hemocytometer. The cell number was confirmed by determining colony-forming units per mL (CFU/mL) on YPD plates.

### Inoculation of *G. mellonella* with *C. albicans* strains

*G. mellonella* (Vanderhorst Wholesale, St. Marys, OH, USA) in the final larval stage were stored in the dark and used within 7 days from shipment. Sixteen randomly chosen *G. mellonella* larvae with similar weight and size (250-350 mg) were used per group in all assays. Two control groups were included: one group was inoculated with PBS to observe the killing due to physical trauma, and the other received no injection as a control for general viability.

A Hamilton syringe was used to inject 5 μL inoculum aliquots into the hemocoel of each larvae via the last left *proleg* containing 10^6^ CFU/larvae of *C. albicans* cells suspended in PBS. After injection, larvae were incubated in plastic containers at 37°C and monitored for survival daily.

### Chemicals and photosensitizer

Methylene blue (MB, Sigma, St Louis, MO) was used at a final working concentration of 1 mM. The dye was dissolved in distilled and deionized filter sterilized water (ddH_2_O). For each experiment, a new PS solution was prepared daily. Fluconazole (Sigma-Aldrich, Steinheim, Germany) was dissolved in ddH_2_O and injected in *G. mellonella* at a concentration of 14 mg/Kg.

### Antimicrobial photodynamic therapy

The *G. mellonella* larvae were injected with 10 μL of a 1 mM solution of MB 90 min after the *Candida* infection and the PS was allowed to disperse for 30 min into the insect body in the dark, prior to the light irradiation.

A broad-band non coherent light source (LumaCare, Newport Beach, CA) was used for light delivery. This device was fitted with a 660 ± 15 nm band-pass filter probe that was employed to produce a uniform spot for illumination. The optical power was measured using a power meter (PM100D power/energy meter, Thorlabs, Inc., Newton, NJ).

### Antifungal administration

For the study of aPDT combined with conventional antifungal drug, fluconazole (14 mg/kg) was injected immediately before or after the exposure of larvae to light. As a control, a group of the larvae received an injection containing PBS, in lieu of fluconazole.

### *G. mellonella* survival assays

After aPDT or combined treatment of aPDT with fluconazole, larvae were observed every 24 h, and considered dead when they displayed no movement in response to touch. Survival curves were plotted and statistical analysis was performed by the Log-rank (Mantel-Cox) test using Graph Pad Prism statistical software. A *P* value <0.05 was considered statistically significant. All experiments were repeated at least twice, representative experiments are presented.

### Persistence of *C. albicans* in the hemolymph of *G. mellonella*

The number of fungal cells recovered from the hemolymph of *G. mellonella* infected by *C. albicans* Can37 was measured immediately after larvae were exposed to aPDT and to combined treatment (aPDT and fluconazole). Three surviving larvae per group were bled by insertion of a lancet into the hemocoel. Hemolymph from 3 larvae was pooled into 1.5 ml Eppendorf tubes in a final volume of approximately 80 μL. Then, the hemolymph was serially diluted and plated on Sabouraud dextrose agar supplemented with chloramphenicol (100 mg/L). Plates were incubated aerobically at 37°C for 24 h, and colonies were counted in each pool (CFU/pool). The groups exposed to aPDT were compared to the control groups by Student *t* test. Difference in the number of CFUs were considered statistically significant at *P* < 0.05. The experiments were repeated at least twice and representative experiments are presented. Three polls per group were performed in each experiment.

## Results

We previously described the utility of the *G. mellonella* model host to assess antibacterial PDT efficacy against *E. faecium*[19]. In this study we explored the potential of this model using antifungal therapy against one of the most common opportunistic fungal pathogens *C. albicans*. Briefly, after 90 min of *Candida* infection, *G. mellonella* larvae were treated with PDT mediated by MB and red light according to the methods described.

As a first step in exploring the optimal dose–response to MB mediated-PDT, we evaluated 10 groups of larvae that were infected with the wild-type strain of *C. albicans* (Can14) and received MB (1 mM) injection. We gradually increased the light exposure time. More specifically, eight groups were exposed to red light at different fluences (0.9, 1.8, 3.6, 5.4, 7.2, 10.8, 14.4 and 18 J/cm^2^, corresponding to 30, 60, 120, 180, 240, 360, 480 and 600 s of irradiation), while two control groups received injection of PBS or MB with no light exposure. After irradiation, the survival rate of *G. mellonella* was assessed 24 h post *C. albicans* infection. The best survival rate was reached with the lowest dose and 30 s of irradiation time (data not shown).

**Figure 1:**
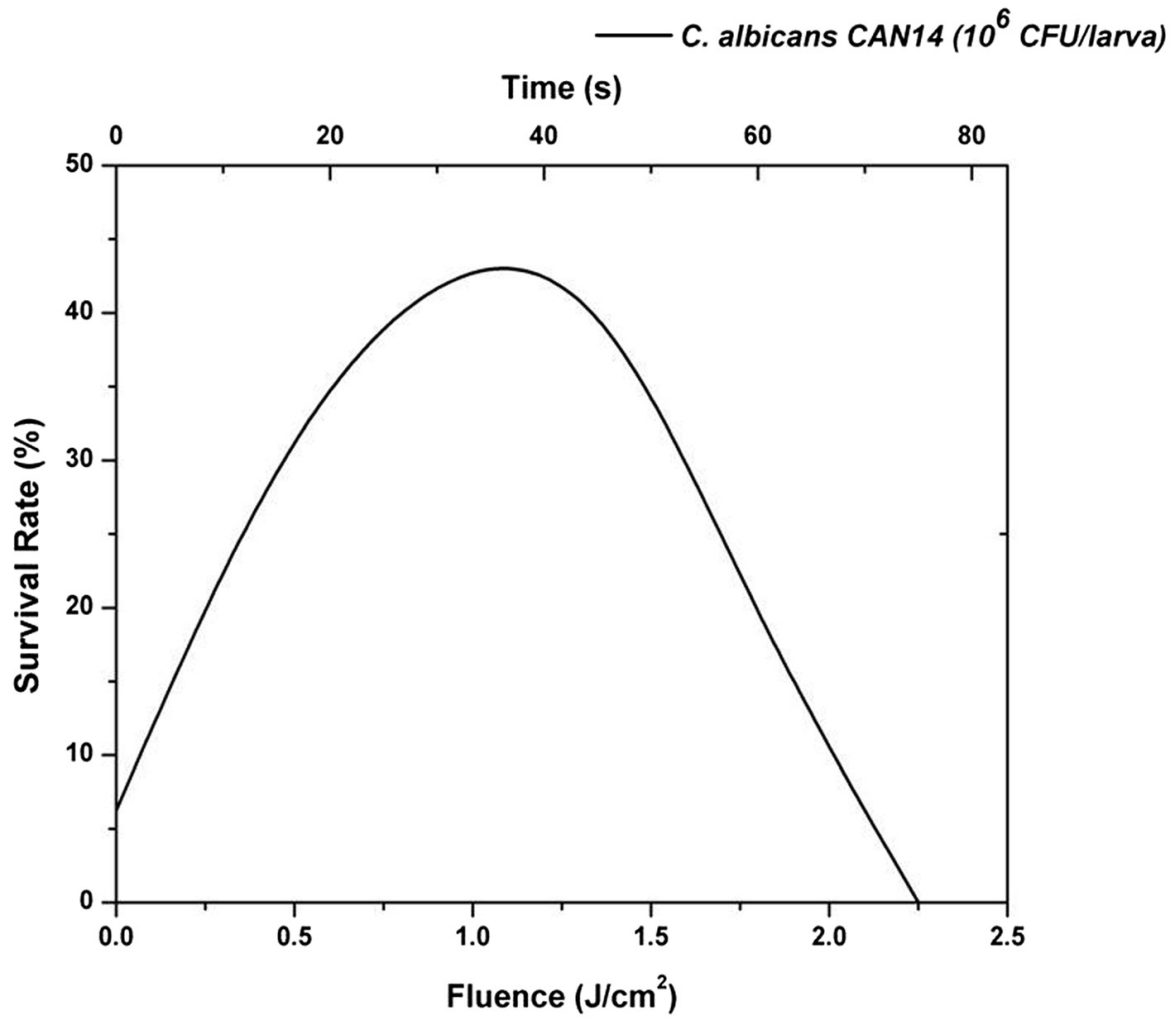
**Dose–response 24 h after aPDT in *G. mellonella *infected by *C. albicans *Can14.** Larvae were infected with 1x10^6^ CFU/larva of *C. albicans* Can14. The best survival rate was found when the fluence of 0.9 J/cm^2^ was applied.

As a second step, a finer evaluation to establish the optimum light dosimetry was performed. Eight further groups were employed to analyze the photodynamic effects at 15, 30, 45, 60, 75, 90, 105 and 120 s of irradiation (0.45, 0.9, 1.35, 1.8, 2.25, 2.7 and 3.6 J/cm2) and once again 0.9 J/cm^2^ (30 s of irradiation) provided the best survival rate (Figure 1).

**Figure 2:**
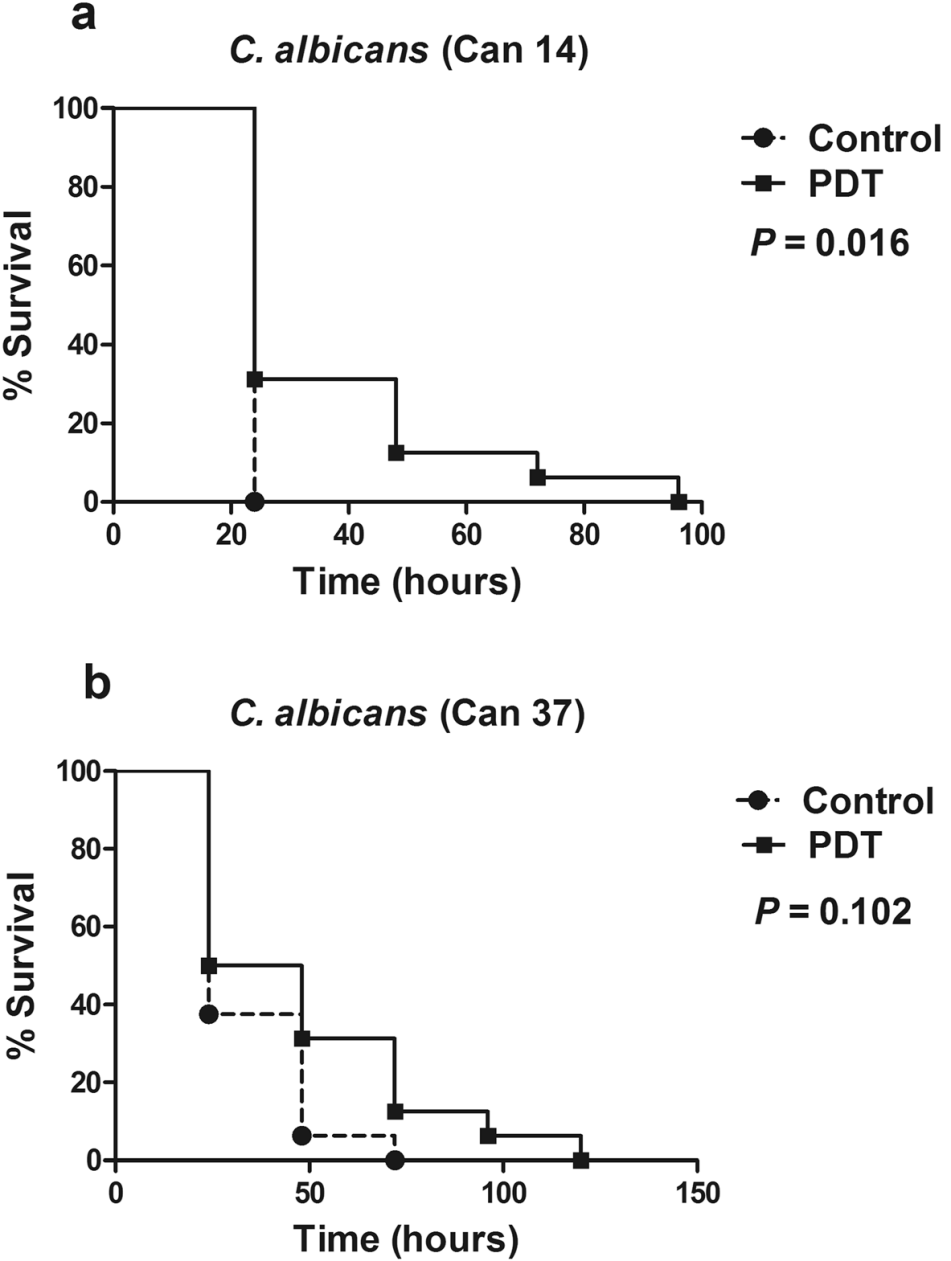
**Killing of *G. mellonella *by *C. albicans *exposed to antimicrobial PDT.** In the aPDT group, the larvae received the PS injection 90 min after the infection with *C. albicans*. In order to allow a good dispersion of the PS into the insect body, we waited at least 30 additional min after the PS injection prior to the light irradiation. A control group received PS without light exposure. Larvae were maintained at 37°C. **a)***C. albicans* Can14 wild-type strain SC5314, **b)***C. albicans* Can37 clinical isolate from oropharyngeal candidiasis and fluconazole resistant.

As a third step, a further comprehensive experimental procedure was designed to assess the effects of aPDT, mediated by the optimum dose (1 mM MB and red light at 0.9 J/cm^2^), on host curve survival when infected by the wild-type strain *C. albicans* Can14 and the fluconazole resistant isolate *C. albicans* Can37. We observed that MB-mediated aPDT, prolonged the larval survival when compared to non-PDT treated larvae, however a statistically significant difference between PDT and control groups was observed only for *C. albicans* Can14 (Figure 2).

**Figure 3:**
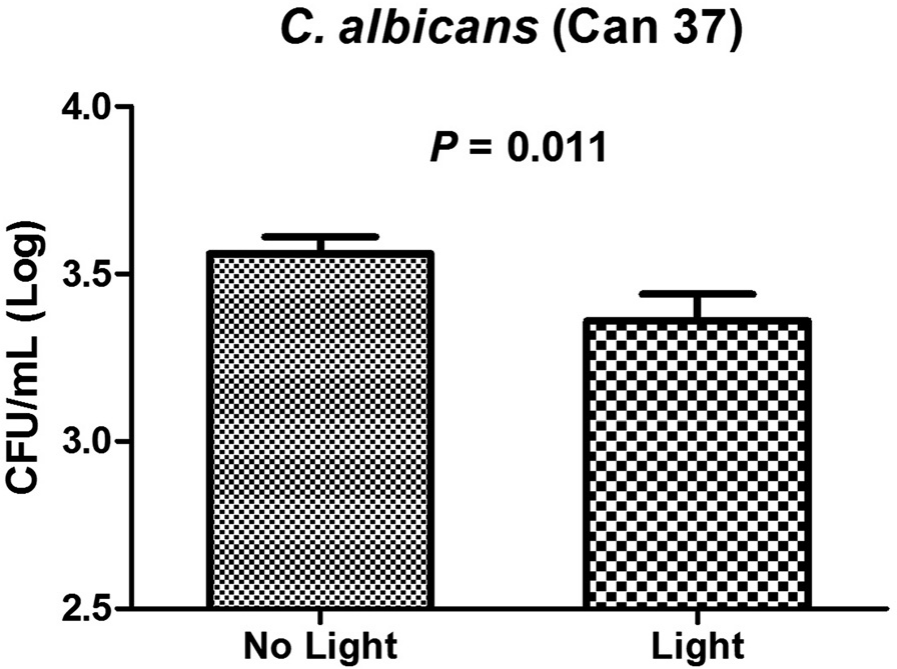
**Number of fungal cells in *G. mellonella *hemolymph immediately post exposed to antimicrobial PDT treatment.** Larvae were infected with 1.41x10^6^ CFU/larva of *C. albicans* Can37 and were maintained at 37°C. After 90 min post-infection, the PS was injected. We waited an additional 30 min prior to light irradiation. After light irradiation, the bacterial burden was measured immediately. Fungal burden was quantified from pools of three larvae hemolymph. aPDT exposed groups resulted in a significant fungal burden reduction when compared to the control group that was not exposed to light. Bars and error bars represent, respectively, the mean and standard deviation of three pooled larvae per group.

Since it was observed that fluconazole resistant strain (Can37) showed reduced sensitivity to PDT, we evaluated the number of CFU within the hemolymph to determine if the fungal burden was reduced even if survival was not significantly increased. We compared the hemolymph burden of aPDT-treated larvae with non-treated larvae and a significant reduction in the CFU number was observed post-PDT treatment (Figure 3). These results confirmed that aPDT was able to reduced fungal cell viability (0.2 Log) immediately upon light exposure, suggesting that singlet oxygen and other ROS were produced, leading to cell damage [21,22].

**Figure 4:**
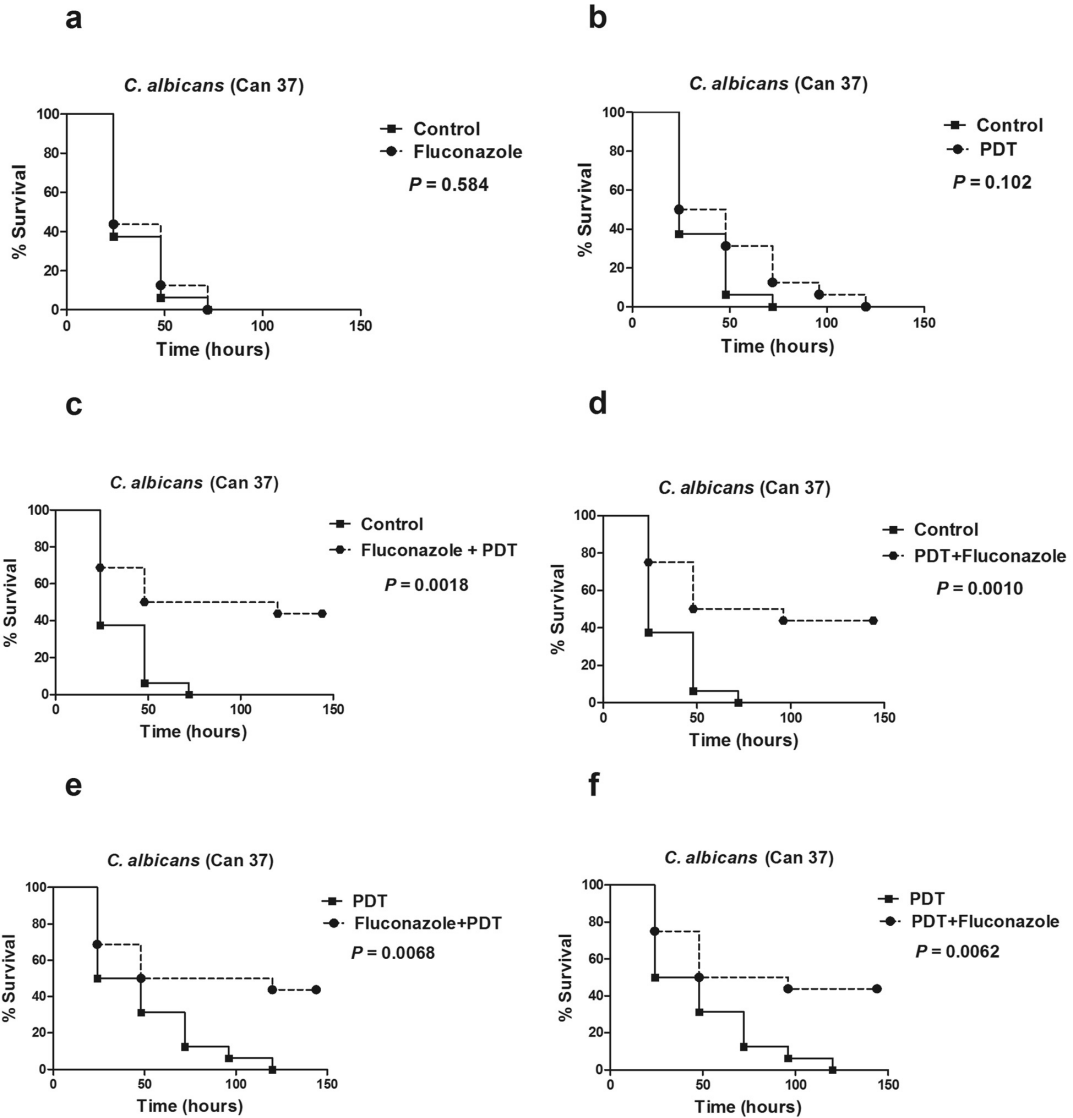
**Killing of *G. mellonella *larvae after infection by *C. albicans *Can37 fluconazole resistant.** The larvae received an injection of 1.4x10^6^CFU/larva and were maintained at 37°C. **a)** administration of fluconazole (14 mg/kg) or PBS (Control), **b)** antimicrobial PDT or only MB (Control), **c)** administration of fluconazole followed by aPDT in a combined therapy or PBS (Control), **d)** administration of aPDT followed by fluconazole in a combined therapy or PBS (Control), **e)** administration of aPDT or fluconazole + PDT, **f)** administration of aPDT or fluconazole + PDT. There was no significant difference on larvae survival when treatment was done only by injecting of fluconazole (*P* = 0.584) or aPDT alone (*P* = 0.102). The combined treatment by application of aPDT followed or before fluconazole injection resulted in significantly lower death rates when compared to a control groups (*P* = 0.0010 to aPDT followed by fluconazole, and *P* = 0.0018 when aPDT was applied after fluconazole injection). A significant difference in survival was observed for combined treatment compared to aPDT alone (*P* = 0.0062 for aPDT followed by fluconazole, and *P* = 0.0068 when aPDT was applied after fluconazole injection).

The reduced fungal burden indicates that the aPDT treated cells are potentially damaged and thus the survival might be altered by the addition of another cell membrane directed bombarding compound, a structure important for the maintenance of cell wall integrity. Hence, we investigated the effects of combined treatment of aPDT with fluconazole, a compound that targets P450 and affects ergesterol synthesis, a major component of the cell membrane. This antifungal agent is used extensively because of its low host toxicity to treat fungal infections. One of the mechanisms that can be used by *C. albicans* to develop resistance to fluconazole is related to the overexpression of cell membrane multidrug efflux systems [23,24]. Based on the hypothesis that aPDT could damage the cell membrane of *C. albicans*, producing increased membrane permeability [25] and possibly damaging efflux pumps, we used *G. mellonella-C. albicans* system to assess the sequential combination of PDT with fluconazole. *G. mellonella* were inoculated with 1.41 × 10^6^ CFU/larva to infect the larvae with the fluconazole-resistant *C. albicans* strain (*C. albicans* Can37). Larvae treated only with PDT or only with fluconazole did not show significantly prolonged larval survival. The sequential combination with fluconazole, before or after PDT, significantly increased larvae survival in both assays (Figure 4). These results suggest that aPDT increases the susceptibility of *C. albicans* Can37 to fluconazole.

## Discussion and conclusion

In this study we used the invertebrate model *G. mellonella* for the *in vivo* study of antifungal PDT. We verified that aPDT prolonged the survival of *G. mellonella* caterpillars infected by *C. albicans* and reduced the fungal burden in the hemolymph of these animals. In addition, we used a fluconazole-resistant *C. albicans* strain to test the combination of aPDT and fluconazole. The data presented here demonstrated that aPDT increased the susceptibility of *C. albicans* to fluconazole.

The increased numbers of fungal infections and the subsequent need for high-cost and time-consuming development of new antimicrobial strategies and anti-infectives has emerged as a major problem among infectious diseases researchers and clinicians [6,26]. Antimicrobial PDT is one of the most promising alternative countermeasures for cutaneous or mucosal infections, caused by either bacteria or fungi [6,26].

Antifungal PDT is an area of increasing interest, as research is advancing in answering fundamental questions regarding the photochemical and photophysical mechanisms involved in photoinactivation; producing new, potent and clinically compatible PS; and in understanding the effect of key microbial phenotypic multidrug resistance, virulence and pathogenesis determinants in photoinactivation. The novel concept of developing the non-vertebrate infection model in *G. mellonella* to explore the efficacy of antifungal PDT provides many competitive advantages [6].

The use of the invertebrate model host has significant benefits when compared to mammalian animals: there are no ethical or legal concerns, no need for specialized feeding or housing facilities, the management of the animal is very easy and no anesthesia is needed, animals are inexpensive, and the use of large sample numbers in the same group are possible [27-30]. *G. mellonella* has been used to study host-pathogen interactions as an alternative host model to small mammals such as mice and rats [9,27-29,31-40].

Our laboratory pioneered the use of *G. mellonella* as a suitable invertebrate model host to study aPDT against *Enterococcus faecium*[19]. In the present study this approach to investigating aPDT was successfully expanded to include fungal pathogens. The optimal dose–response to MB mediated-PDT was evaluated and 0.9 J/cm^2^ showed the best survival of *G. mellonella* caterpillars, as was found in the *E. faecium* study. The same limited non-toxic dosage of aPDT to *G. mellonella* was applied to treat larvae infected by strains of *Candida albicans*.

During the *G. mellonella* killing assays, groups infected by *C. albicans* that received aPDT treatment demonstrated prolonged survival when compared to groups that did not received treatment. However a statistically significant difference between PDT and control groups was observed only for *C. albicans* Can14 wild-type strain. When the infection was induced by a fluconazole resistant strain (Can37), a statistically significant difference between these groups was not observed. Despite the fact that PDT has been described as a potent agent against both antimicrobial-resistant and sensitive microorganisms [6] we observed that a fluconazole-resistant *C. albicans* strain was less sensitive to aPDT.

This difference has also been described in an *in vitro* study performed by Dovigo et al. [41]. These authors observed that fluconazole-resistant strains of *C. albicans* and *C. glabrata* showed reduced sensitivity to aPDT in comparison with reference strains susceptible to fluconazole, suggesting that resistance mechanisms of microorganisms to traditional antifungal drugs could reduce PDT effectiveness. According to Prates et al. [23], the resistance of *Candida* strains to fluconazole usually involves overexpression of cell membrane multidrug efflux systems belonging to the ATP-binding cassette (ABC) or the major facilitator superfamily (MFS) classes of transporters. The authors showed that the overexpression of both systems reduced MB uptake by fungal cells, as well as the killing effect of aPDT, suggesting that ABCs and MFSs are involved in the efficiency of aPDT mediated by MB and red light. In addition, Arana et al. [42] demonstrated that subinhibitory concentrations of fluconazole induced oxidative stress and a transcriptional adaptative response that was able to generate protection of *C. albicans* against subsequent challenges with oxidants. The mechanisms of protection against oxidative stress of fluconazole resistant *C. albicans* strain may have enhanced the resistance of *C. albicans* to oxidative damage caused by PDT.

In this study, we also evaluated the effects of aPDT on fungal cells in the hemolymph of *G. mellonella* larvae infected by fluconazole resistant *C. albicans* (Can37). Although this *C. albicans* strain had not shown a significant increase in survival rate in *G. mellonella*, it was observed that aPDT caused a reduction of the number of fungal cells in the hemolymph (0.2 Log) with a statistically significant difference between aPDT and control groups. In addition, these data demonstrated that aPDT was able to reduce fungal cell viability immediately upon light exposure, suggesting that *C. albicans* cells were sensitive to aPDT, by the lethal oxidative damage of the singlet oxygen pathway, in the experimental candidiasis in the *G. mellonella* model. At the moment, all the aPDT studies performed *in vivo* were developed in vertebrate models of rats and mice using fluences of light much higher than the dose used in our work [43-45]. Using an oral candidiasis mice model, Costa and colleagues [44] found a reduction of 0.73 Log in the fungal cells recovered after erythrosine- and LED-mediated aPDT when a fluence of 14 J/cm^2^ was applied. Dai et al. [45] also demonstrated that aPDT, with the combination of methylene blue and red light (78 J/cm^2^), reduced (0.77 Log of CFU) the fungal burden in skin abrasion wounds in mice infected with *C. albicans*.

Patients with fungal infections are often treated with azole antifungal drugs, however *Candida* resistance to azoles has been detected in recent years. Several mechanisms of resistance have been reported including the overexpression of cell membrane multidrug efflux pumps previously cited, an alteration in the chemical structure of the demethylase enzyme, and the incorporation of alternative sterols to ergosterol within the cell membrane [23,24]. Giroldo et al. [25] suggested that MB-mediated aPDT caused damage to the cell membrane of the *C. albicans* cells. If the hypothesis that aPDT could affect the cell membrane is valid, the sequential use of aPDT with fluconazole could have a dual action on treating the infection. Conventional antimicrobial therapy could have aPDT as an adjunct or as an alternative [15]. The combination of PDT with antimicrobials has been used with success when compared to either isolated approach [19,26,46]. Kato et al. [43] verified that after exposure to sublethal aPDT, the minimal inhibitory concentration (MIC) of fluconazole against *C*. *albicans* was reduced compared to non-aPDT treated strains.

Of note, we observed that the *G. mellonella* larvae survival after infection by the fluconazole resistant *C. albicans* strain, was prolonged when fluconazole was administered before or after aPDT, in comparison to the use of fluconazole or PDT alone. We believe that due to the permeabilization of the fungal cell membrane by the sublethal PDT dose, fungal cells become more susceptible to fluconazole action. In addition, it has been suggested that the use of azoles can increase the oxidative stress promoted by PDT by contributing to ROS formation themselves [26]. Arana et al. [42] demonstrated that fluconazole was able to induce oxidative stress in *C. albicans* in a dose- and time-dependent manner, suggesting that ROS play a role in the mechanism of action of azoles. The exact mechanism involved in increasing the survival of larvae infected by the fluconazole resistant *C. albicans* strain and exposed to combined therapy of PDT and fluconazole remains to be clarified. Thus, comprehensive experiments are needed to better understand whether this process could be useful to treat antimicrobial resistant fungal infections.

In summary, the results obtained in this study showed that *G. mellonella* is a suitable model host to study the antifungal PDT *in vivo*. It is known that the *G. mellonella* model is not restricted to studies that examine aspects of the pathogenesis of fungal infections or antimicrobial therapies, but also can be used to the study of host defenses against fungal pathogens [30]. The insect immune response demonstrates a number of strong structural and functional similarities to the innate immune response of mammals and, in particular, insect haemocytes and mammalian neutrophils have been shown to phagocytose and kill pathogens in a similar manner [47]. Recent studies demonstrated that PDT can stimulate host defense mechanisms. Tanaka et al. [21] used a murine methicilin-resistant *Staphylococcus aureus* (MRSA) arthritis model and verified that the MB-mediated PDT exerted a therapeutic effect against a bacterial infection via the attraction and accumulation of neutrophils into the infected region. Neutrophils are among the first cells recruited to the illuminated area and their main function is to release enzymes for killing infectious organisms and secrete cytokines and other chemicals that promote inflammation [48]. In this study, the effects of aPDT on the immune system of *G. mellonella* were not investigated. Therefore, future studies need to be developed to understanding the action of aPDT and methylene blue in the haemocyte density and in the expression of a variety of antimicrobial peptides involved in immune responses of *G. mellonella*.

The key conclusion is that the *G. mellonela - C. albicans* system is a suitable model to study antifungal PDT and to explore combinatorial aPDT-based treatments. Thus, this invertebrate animal model host provides a novel approach to assess the effects of *in vivo* PDT, alone or in combination with antifungal compounds, on fungal infections without the difficulties of mammalian models.

## Authors’ contributions

Conceived and designed the experiments: JCJr, CPS, XT, BBF, MRH, EM. Performed the experiments: JCJr, CPS, XT, YW. Analyzed the data: JCJr, JCJ, AOCJ, GPT, MRH, EM. Contributed reagents/materials/analysis tools: MRH, EM. Wrote the paper: JCJr, JCJ, MRH, GPT, EM. All authors read and approved the final manuscript.
